# Acetoacetate reduces growth and ATP concentration in cancer cell lines which over-express uncoupling protein 2

**DOI:** 10.1186/1475-2867-9-14

**Published:** 2009-05-29

**Authors:** Eugene J Fine, Anna Miller, Edward V Quadros, Jeffrey M Sequeira, Richard D Feinman

**Affiliations:** 1 Department of Nuclear Medicine, Albert Einstein College of Medicine, Bronx, New York, USA; 2 Department of Medicine, SUNY Downstate Medical Center, Brooklyn, New York, USA; 3 Department of Biochemistry, SUNY Downstate Medical Center, Brooklyn, New York, USA

## Abstract

**Background:**

Recent evidence suggests that several human cancers are capable of uncoupling of mitochondrial ATP generation in the presence of intact tricarboxylic acid (TCA) enzymes. The goal of the current study was to test the hypothesis that ketone bodies can inhibit cell growth in aggressive cancers and that expression of uncoupling protein 2 is a contributing factor. The proposed mechanism involves inhibition of glycolytic ATP production via a Randle-like cycle while increased uncoupling renders cancers unable to produce compensatory ATP from respiration.

**Methods:**

Seven aggressive human cancer cell lines, and three control fibroblast lines were grown in vitro in either 10 mM glucose medium (GM), or in glucose plus 10 mM acetoacetate [G+AcA]. The cells were assayed for cell growth, ATP production and expression of UCP2.

**Results:**

There was a high correlation of cell growth with ATP concentration (r = 0.948) in a continuum across all cell lines. Controls demonstrated normal cell growth and ATP with the lowest density of mitochondrial UCP2 staining while all cancer lines demonstrated proportionally inhibited growth and ATP, and over-expression of UCP2 (p < 0.05).

**Conclusion:**

Seven human cancer cell lines grown in glucose plus acetoacetate medium showed tightly coupled reduction of growth and ATP concentration. The findings were not observed in control fibroblasts. The observed over-expression of UCP2 in cancer lines, but not in controls, provides a plausible molecular mechanism by which acetoacetate spares normal cells but suppresses growth in cancer lines. The results bear on the hypothesized potential for ketogenic diets as therapeutic strategies.

## Background

Otto Warburg observed that many cancers lose their capacity for mitochondrial respiration, limiting ATP production to anaerobic glycolytic pathways [1]. The phenomenon is particularly prevalent in aggressive malignancies, most of which are also hypoxic. Hypoxia induces a stochastic imbalance between the number of reduced mitochondrial species *vs*. available oxygen, resulting in increased reactive oxygen species (ROS) whose toxicity can lead to apoptotic cell death. One mitochondrial adaptation to increased ROS is over-expression of uncoupling protein 2 (UCP2) which has been reported in a number of human cancer cell lines [2-4]. Horimoto et al. [3] demonstrated UCP2 over-expression in most of the 120 colon cancer lines tested, the extent correlating with the degree of tumor aggressiveness. Increased UCP2 expression was also associated with reduction in ATP production in malignant oxyphilic thyroid tumors [2], and in mouse leukemia and human lymphoma cell lines [4]. It is reasonable to ask whether one can take advantage of the ability of UCP2 to disrupt regulation of ATP generation in cancer as a therapeutic method.

One mechanism by which aggressive cancers that over-express UCP2 might inhibit glycolytic ATP is the so-called Randle cycle. In normal cells, the Randle cycle has been proposed as a mechanism by which increased availability of fatty acids and ketone bodies from lipolysis inhibit glycolysis in order to maintain stable production of ATP. Products of lipolysis, according to this mechanism, supply acetyl-CoA for the TCA cycle whose intermediates inhibit glycolysis [5]. We propose that aggressive cancers, under conditions of ketosis, employ a Randle-like cycle to inhibit glycolytic ATP generation, but, in contrast to normal cells, cannot supply compensatory mitochondrial ATP due to uncoupling.

We have cultured three human primary fibroblast lines and seven human cancer cell lines in media of varying glucose and ketone body content to compare cell growth rates, ATP concentration, and expression of UCP2. Restriction of cancer cell lines to colon and breast was meant to parallel the kinds of patients we expect to be eligible for our ongoing clinical trial of very low carbohydrate diets in advanced cancer[6]. (The trial is registered with ClinicalTrials.gov; Registry No. NCT00444054; with information available at ).

## Materials and methods

### Cell lines

Human cell lines representing colon cancers (Lovo, RKO, CaCO2, SW48, SW480) and breast cancers (MDA MB 231, MCF7) were purchased from ATCC, and control lines of normal human fibroblasts RFP3, MCH 064 and MCH 065 were obtained from other sources. Cells were cultured in DMEM with 10% fetal calf serum and 10 mM glucose in 4% CO_2 _at 37°C. Cells were plated into twelve well plates at a density of 10^5 ^per ml into their respective media and were maintained for 24, 48, 72, and 96 h to determine their growth curves. For cells maintained in culture of 72 and 96 h, medium was replaced at 48 h. Cells were harvested by trypsinization and cell count and viability were determined in the presence of trypan blue dye using a hemocytometer. Glucose concentrations were varied from 5 mM, 10 mM, 25 mM, 50 mM and 100 mM to determine optimum growth conditions. All plated cell lines were incubated in duplicate or triplicate.

### Materials

Modified DMEM without glucose, glutamine, and pyruvate, 100× NEAA and Lithium acetoacetate (Sigma), fetal bovine serum (GIBCO) 100× glutamine, 100× pyruvate, Trypsin: EDTA solution, normal goat serum, 100× antibiotic solution and ATP determination kit (Gibco/Invitrogen); rabbit anti-UCP2 (human-Biolegend); goat anti-rabbit IgG (H+L)-HRP conjugate (Vector Labs).

### Assays

#### Cellular ATP and protein content

ATP content in the cells was assayed using a luciferase assay kit (Sigma). Total protein was assayed using the Biorad protein assay kit and the ATP content was expressed per cell, and per mg of cellular protein.

#### Immunohistochemistry

Cells grown in 12 well plates for 96 h were dissociated with 0.5 ml Trypsin:EDTA solution, re-suspended in equal volume of DMEM containing 10% FBS, washed twice in one ml PBS, followed by centrifugation at 2800 rpm for 3 min. Cells were re-suspended in 4 volumes of PBS, spread on aminosilane coated slides and air dried overnight. Doubly PBS-washed slides were blocked with 10% normal goat serum in PBS for 30 min. at room temperature. The slides were then incubated in rabbit anti-UCP2 (human) Ab diluted 1:200 in 10% normal goat serum-PBS for 2 h at room temperature or overnight 4°C. Slides washed in PBS were incubated in 1:2000 dilutions of goat anti-rabbit IgG (H+L)-HRP conjugated secondary Ab in 10% normal goat serum -PBS for 1 h at room temperature then washed in PBS again. Slides were then incubated in DAB peroxidase substrate for 6–10 minutes, washed in water, dehydrated through alcohols and xylenes and covered with permount medium and glass cover slip.

#### Quantitation of UCP2 immunostaining using ImageJ

All cell types placed on a slide were simultaneously immunostained for UCP2 as previously described and photographed at 100× magnification using a Nikon Labophot-2 microscope equipped with a Nikon D70 digital camera. The relative intensity of UCP staining per cell was determined using ImageJ  version 1.39 (NIH, USA: for latest supported version see ).

Briefly, RGB composite images containing both control cells (normal human fibroblasts) and various cancer cell lines were created using Photoshop CS. The composite files were opened within ImageJ and converted to 32 bit grayscale images. Following this conversion the threshold for analysis was automatically chosen for the entire composite image by ImageJ. A frame was then drawn around each cell type in the composite image and a particle analysis was performed that generated data containing both the cell count and the intensity of stain per cell. The frame was then moved to another cell type within the composite image and the analysis re-performed.

All UCP2 results were reported using the immunostain method with ImageJ analysis of optical density.

#### Specificity of immunostain and quantitation of UCP2 with ELISA sandwich assay

An ELISA sandwich assay was developed that was directed against widely separated peptide fragments of UCP2. Details of preparation will be published elsewhere. In brief, UCP2 antigen and rabbit anti-human UCP2 as primary antibody (recombinant N-terminal partial fragment) were obtained from Biolegend. A secondary antibody for the sandwich assay was Goat anti-rabbit HRP H&L from Vector Labs. TMB was reacted with the peroxidase and absorbance was measured at 450 nm. Results of UCP2 by ELISA were correlated with ImageJ analysis for RFP3, SW48, and MDA MB 231 to assess specificity of the immunostain method.

### Statistics

Correlation of ATP/mg protein with cell growth was performed using linear least squares regression fit. UCP2 OD and ATP/mg protein were compared among all cell lines using two-way ANOVA. Correlations at p < 0.05 level were considered significant. Comparisons of UCP2 in cancer lines vs. mean fibroblast values were performed by the Student t-test with Bonferroni correction.

## Results

Preliminary examination of cell culture of all lines demonstrated a peak in cell growth rates at either 10 mM glucose or 25 mM glucose compared with higher or lower concentrations. 50 mM and 100 mM glucose suppressed cell growth, likely from hypertonicity. Ninety-six h permitted maximum growth before plate saturation and was selected as the optimal time for cell counting. It was found that 10 mM glucose medium (GM) was as effective at promoting growth as 25 mM and, because it is closer to physiologic human concentrations, was chosen for further experimental testing.

In order to compare the effect of acetoacetate (AcA) on growth, cell counts at 96 h in the combined glucose plus acetoacetate medium (G+AcA) were compared to 96 h G alone which was defined as 100%. Table 1 shows values for cell growth by this definition and in absolute cell numbers. Values are also shown for ATP/mg cell and UCP2 as optical densities from scanned photographs of cells stained with peroxidase.

**Table 1:** 96 h cell lines grown in 10 mM glucose either with or without acetoacetate*

**Cell line**								
[Acetoacetate]^d^	ATP^e ^nmols/mg protein	%ATP^f^	Cell count ×10^6^/ml	%cell count^g^	UCP2 OD	% viability	Total Protein/10^6 ^cells	% total Protein

**RFP3**								

*0	85.2	100%	0.85	100%	66.0	99.7	9.85	100%

*10 mM	90.2	106%	0.805	94.7%	47.7	99.7	10.63	107.9%

**MCH 064**								

*0	146.3	100%	0.126	100%	104.5	94.0	7.61	100%

*10 mM	185.8	127%	0.140	111.1%	69.7	98.2	8.80	115.6%

**MCH 065**								

*0	198.5	100%	0.198	100%	80.0^h^	98.8	8.75	100%

*10 mM	184.5	93.2%	0.206	101.5%	59.9^h^	97.0	9.45	108.0%

**SW48**								

*0	693.5	100%	1.94	100%	***177.7***	99.4	7.8	100%

*10 mM	**303.5**	**43.8%**	**0.76**	**39%**	***109.7***	100	7.7	98.7%

**SW480**								

*0	597.0	100%	1.39	100%	***182.7***	95.5	12.8	100%

*10 mM	**428.5**	**71.8%**	**0.79**	**57%**	***161.8***	93.5	11.6	90.6%

**RKO**								

*0	700.0	100%	5.40	100%	***221.1***	100	14.6	100%

*10 mM	**371.0**	**53%**	**2.74**	**50.7**	***181.5***	100	12.6	86.3%

**MCF-7**								

*0	590.0	100%	0.87	100%	***177.1***	97.0	11.95	100%

*10 mM	540.5	91.6%	**0.65**	**74.7%**	***148.2***	91.5	10.8	90.5%

**LOVO**								

*0	433.0	100%	1.02	100%	***150.5***	98.9	13.1	100%

*10 mM	**293.5**	**67.8%**	**0.60**	**58.7%**	***205.2***	97.9	9.50	68.3%

**CaCO2**								

*0	296.5	100%	0.275	100%	***224.7***	93.5	7.10	100%

*10 mM	**137.0**	**46.2%**	**0.140**	**50.9%**	***295.0***	92.9	7.90	111.3%

**MDA MB 231**								

*0	267.0	100%	0.72	100%	***556.7***	99.1	17.95	100%

*10 mM	**161.5**	**60.5%**	**0.34**	**47.6%**	***341.5***	99.3	21.0	117.0%

*For standard errors of all measurements see Figures 2 – 5 (based upon ATP and cell count studies performed in triplicate; and UCP2 measurements performed in duplicate).

ATP and cell growth values in **bold print **are significantly lower (p < 0.05) than the values in glucose only medium for the same cell line. UCP2 values in ***bold italics ***are significantly higher (p < 0.05) than mean of control fibroblast lines for each growth medium.

^d ^[Acetoacetate] = Concentration of Acetoacetate in medium

^e ^ATP production expressed as nmol ATP/mg protein

^f ^% of ATP production at 96 h compared with 10 mM glucose and 0 mM acetoacetate (defined as 100%)

^g ^Cell count expressed as % of 96 h growth in 10 mM glucose and 0 mM acetoacetate

**Figure 1:**
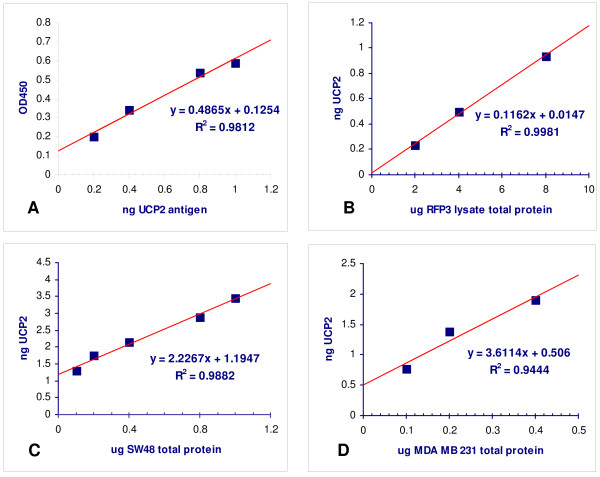
**Specificity of UCP2 ELISA**. (A) Standard curve of UCP2 antigen is calibrated against sandwich ELISA assay (see text). (B) UCP2 is calculated from standard curve in cell lysates from RFP3, SW48 and MDA MB 231 (B, C, and D respectively). Slopes of regressions represent ng UCP2 per μg total protein.

^h ^Calculated from the slope of its ELISA curve (0.043/μg protein, not shown) in comparison to Fig. 1B for RFP3 cells, then recalibrated to ImageJ OD/cell.

### Specificity of assays

**Figure 2:**
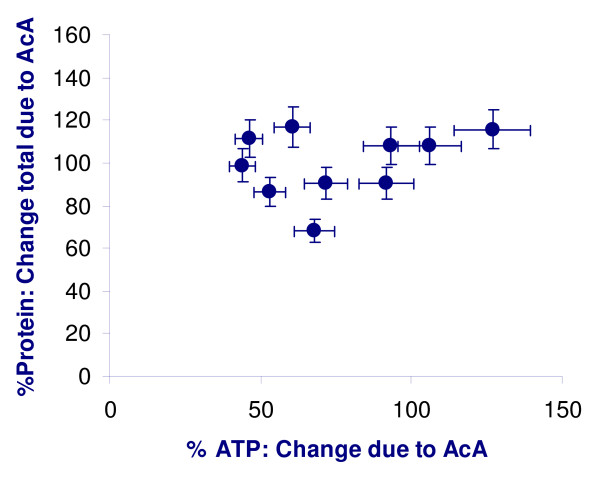
**Lack of non-specific protein inhibition**. There is no significant correlation between expression of cellular protein and the % change in ATP due to supplemental acetoacetate in the medium. r^2 ^= 0.08 (p > 0.2) Data are from Table 1.

The UCP2 ELISA sandwich assay demonstrated a linear relationship for the standard curve of UCP2 antigen (Biolegend) vs. ELISA (r = 0.988) (Figure 1A). Lysates of RFP3, SW48 and MDA MB 231 were also assayed at varying concentrations up to 1000 fold excess total protein. The calculated UCP2 value from the standard curve remained highly linear (Figures 1B, C, and 1D) without evidence of non-specific binding. The UCP2 values in cell lines tested by ELISA remain in the same relation as the immunostain method. ImageJ-derived OD (y) correlates with the ELISA(x) [y = 122.7x + 13.5; r = 0.93, p < 0.01; data not shown]. There was no correlation between the expression of cellular protein and the % change in ATP due to acetoacetate (Figure 2).

### Effects on cell growth and ATP concentration

**Figure 3:**
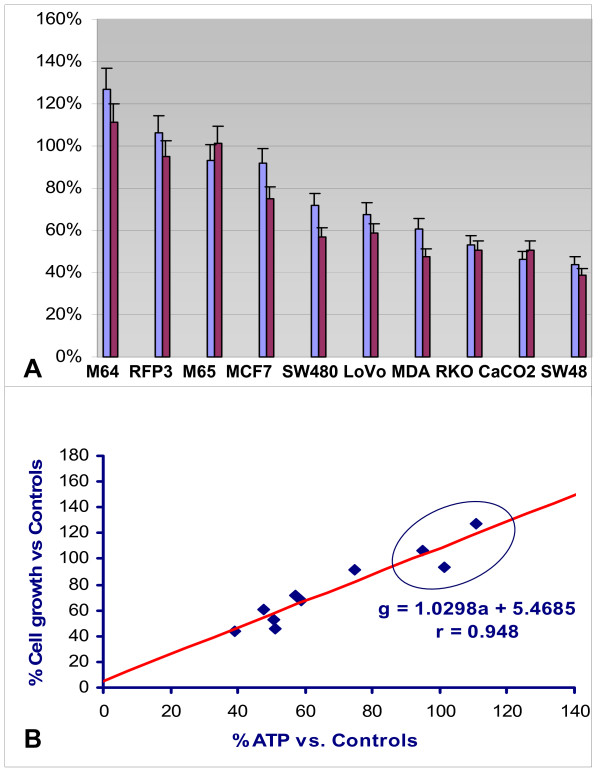
**(A): The effect of adding acetoacetate to glucose medium on ATP concentration and cell growth**. ATP and cell growth are both expressed as a percent of the respective values obtained at 96 h in 10 mM glucose (only) medium. M64 and M65 represent MCH 064 and MCH 065 cell lines, respectively. **(B)** The relation of cell growth to ATP concentration. Normal fibroblast controls RFP3, MCH 064 and 065 (in top right oval) demonstrate neither growth inhibition nor reduced ATP. All other data points represent seven cancer cell lines of Table 1 with parallel reduction in ATP and cell growth as also see in 3A. Regression equation plots growth (g) vs. ATP concentration (a) in blue in lower right of figure (p < 0.01). Data are from Table 1.

Control fibroblasts from a normal adult (RFP3) and from neonates (MH 064 and 065) showed no reduction in either cell growth or ATP concentration in G+AcA medium, whereas all cancer lines showed parallel reductions in ATP and growth when cultured in G+AcA (Table 1 and Figure 3A). ATP reduction and cell growth inhibition show a linear correlation by least squares regression, g = 1.03a +6.5; r = 0.95 (p < 0.001) (Figure 3B).

**Figure 4:**
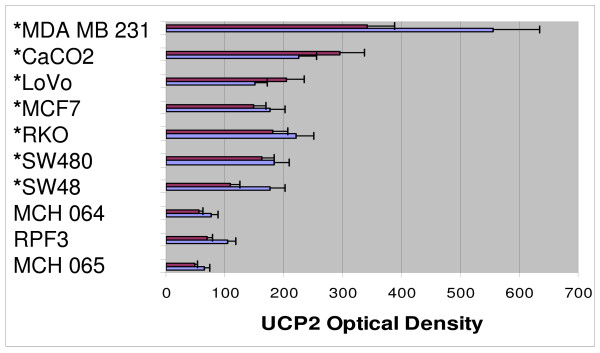
**Cell growth vs. UCP2 expression in fibroblast controls vs. cancer lines**. Purple series represents UCP2 in cells grown in glucose medium; blue series represents cell lines grown in glucose plus acetoacetate. Cancer lines over-express UCP2 vs. control fibroblasts when grown in either medium. Asterisks reflect significant differences (p < 0.05) in UCP2 values for cells grown in G or G+AcA growth medium when compared to corresponding results from all fibroblast lines. Data are from Table 1.

**Figure 5:**
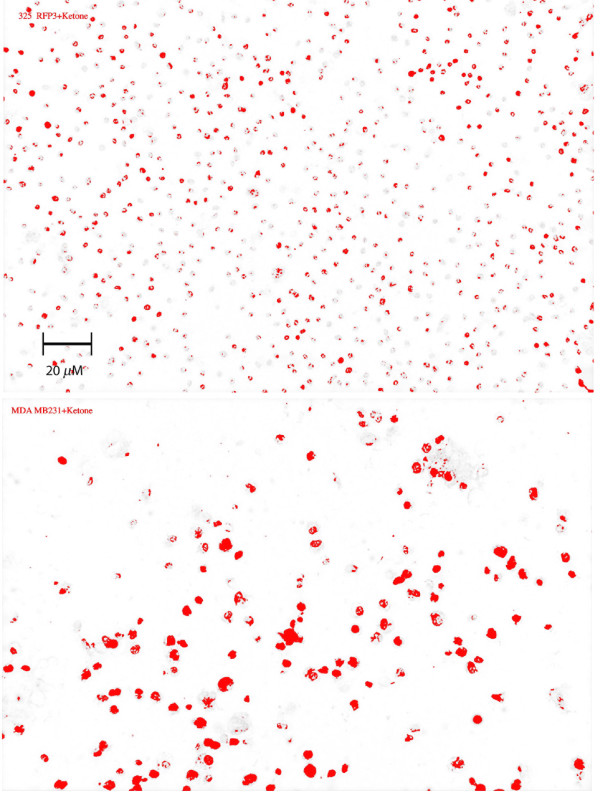
**UCP2 expression in fibroblasts vs. MDA MB 231**. Peroxidase staining of RFP3 controls (top) involves fewer cells and is less intense than of MDA MB 231 (bottom). Magnification 100×.

**Figure 6:**
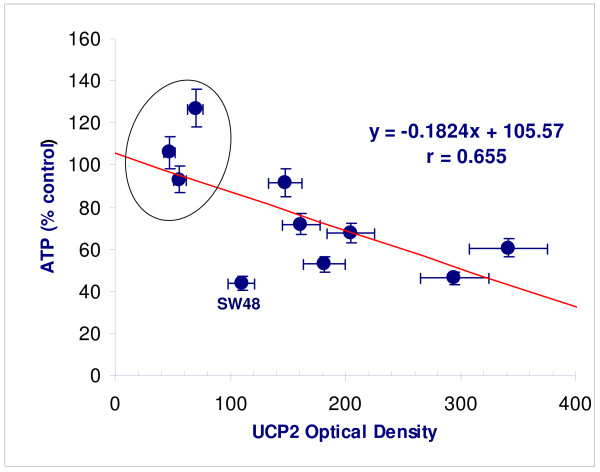
**ATP concentration as a function of UCP2 expression**. (ATP (y) in acetoacetate medium as a function of UCP2 expression (x) [regression equation upper right; p < 0.05]. Lowest expression of UCP2 with greatest ATP preservation is present in control fibroblasts (in oval). SW48 is identified as an outlier. (Data are from Table 1).

UCP2 expression displayed variation at 96 h depending on the growth medium (GM vs. G + AcA), but all cancer lines showed higher values in either medium compared with fibroblasts (p < 0.05) (Figure 4). Peroxidase staining of MDA MB231 is contrasted with RFP3 in Figure 5. There was an inverse linear relation between ATP concentration and UCP2 expression in acetoacetate medium (Figure 6; r = 0.66, p < 0.05).

## Discussion

The uncoupling proteins (UCP's) constitute a structurally related family with variable ability to uncouple mitochondrial production of ATP [7][8,9]. Uncoupling reduces the proton gradient across the inner mitochondrial membrane. UCP1, first discovered in rodents, is present in brown fat in infants but its abundance decreases strikingly in most people with age, along with the reduction of brown fat tissue. UCP2 has about 58% structural homology with UCP1. While UCP2 concentration is very low or absent in most normal tissues it has been found to be more highly expressed in aging vs. young fibroblasts [10], in pancreatic islets in type 2 diabetes mellitus[11], and in macrophages under inflammatory stimulation [7,12]. In all these cases its presence has been postulated to represent an adaptive reaction to increased reactive oxygen species (ROS). Controversy exists whether UCP2 causes significant mitochondrial uncoupling or ROS inhibition [13,14]. It is instructive, however, to note that tissues with the highest UCP2 expression, as above, represent those which generate high ROS; and that ROS mitigation could be explained by mitochondrial uncoupling, an effect observed only at high UCP2 concentrations. Cancers are among the tissues reported to express UCP2 in much higher concentrations than normal tissues [2-4]. Our proposal starts with the assumption that UCP2 over-expression is an adaptive response which limits ROS in cancer lines by causing uncoupling. The proposal asks whether this assumption is borne out with consistent results under specific experimental conditions.

An increase in UCP2 has been reported to be associated with reduction of respiratory ATP production in several cancer lines [2,4] that have preserved TCA cycle enzymes. Horimoto et al [3] showed that UCP2 over-expression is present in *most *colon cancer lines, the degree of over-expression correlating with the histologic grade. It was proposed that UCP2 over-expression was an adaptive mechanism that reduced ROS-induced apoptosis among the cancer cells.

We suggest that ketone bodies, if metabolized in cancers of this type, can inhibit glycoloysis and resultant ATP generation without compensatory mitochondrial respiratory ATP production, thereby inhibiting cell growth.

The Randle cycle was proposed more than 30 years ago as a mechanism whereby products of lipolysis (fatty acids and ketone bodies) would inhibit glycolysis in normal cells to maintain stable ATP production [5]. According to this mechanism, abundant acetyl CoA causes feedback inhibition of pyruvate dehydrogenase and increases the level of TCA intermediates, specifically citrate, to inhibit phosphofructokinase, both processes reducing the rate of glycolysis. The relevance to normal metabolism has been challenged by Wolfe et al [15,16]. These investigators showed convincingly that under most physiologic conditions carbohydrate-stimulated insulin secretion increases intracellular glucose availability. Increased intracellular glucose concentrations drive glycolysis, so that carbohydrate, via the dominating effect of insulin, becomes the regulator of lipid metabolism rather the other way around. Wolfe *et al*. pointed out that the Randle cycle has only been persuasively demonstrated in *in vitro *preparations under conditions of euglycemic, hyperinsulinemic clamp, in which intracellular glucose availability remains high and fixed. Under these circumstances, in rat myocardium preparations, for example, variable fatty acid availability does indeed regulate the rate of glycolysis.

Interestingly, high fixed intracellular glucose availability is a likely condition *in vivo *for most aggressive cancer cells whose membranes express high affinity *insulin-independent *glucose 1 transporters (GLUT1) [17]. The Km for GLUT1, at 18 mg/dl, predicts maximum fixed glucose transport at ordinary physiologic glucose concentrations of 60–100 mg/dl. This contrasts with normal human tissues (e.g. myocytes, adipocytes, endothelial cells) which, capable of metabolizing lipids and carbohydrates, express insulin-dependent GLUT4. Our hypothesis suggests that metabolism of ketone bodies in cells over-expressing GLUT1 and UCP2 may provide the opportunity for acetyl COA and citrate to inhibit glycolysis, just as in the euglycemic-hyperinsulinemic Randle cycle. However, UCP2 over-expression differs from the Randle model in that uncoupling prevents full mitochondrial compensation of ATP production. We consider this an "inefficient" Randle cycle.

Evidence for such an inefficient Randle cycle is found in the seven cancer lines of our investigation which demonstrated parallel reductions in ATP concentration and cell growth, the greatest inhibition found in CaCO2 and SW48 lines, and the least in MCF7 (Figure 3B). In addition, UCP2 staining correlates inversely with ATP production in all cells lines (Figure 4, 5). The three control fibroblast cell lines demonstrated neither growth inhibition nor reduced ATP production, and showed the least quantitative staining for UCP2 among all cell lines measured. The findings extend the observations of previous investigators and point to a specific kind of mitochondrial abnormality, UCP2 over-expression that under conditions of ketosis is capable of reducing the growth rate of certain cancers. We believe that the findings are also capable of reconciling apparently contradictory results in previous animal models. Tisdale *et al*. reported MAC16 tumor growth inhibition in mice on ketogenic diets [18] but failure of ketosis to inhibit Walker carcinosarcoma [19]. The latter tumor lacks the succinyl-CoA ketotransferase required to metabolize ketone bodies. Metabolism of ketone bodies is required by the present hypothesis to enable inhibition of glycolysis via the TCA cycle, and therefore absence of this enzyme would predictably fail to inhibit cancer growth.

There are limitations in the present study. The investigation does not prove metabolic inhibition of cancer growth and ATP production due to uncoupling. Proof requires direct demonstrations of acetoacetate metabolism as well as inhibition of the rate of ATP production, not just of steady state ATP concentrations. Second, glucose and acetoacetate were both incubated at supraphysiologic concentrations to maximize detectable effects. It is not possible without further *in vivo *study to predict effects under physiologic conditions. Third, *in vitro *culture of primary cell lines is difficult for normal human cell types, and only fibroblasts were used as controls, so the response of other normal tissue types was not measured. Further, fibroblast lines capable of growth in cell culture are not normal; however, neither are they the equivalent to malignant cell lines and their difference from malignant lines was demonstrated in this investigation. Also, the range of tumor susceptibility to acetoacetate varies widely, plausibly due to increased levels of UCP2 expression, but other factors must be considered. For example SW48, with the greatest reduction of ATP and cell growth in acetoacetate medium, was a prominent outlier with the least UCP2 expression among the cancer cell lines (Figure 5). Finally, we did not manipulate UCP2 expression using knock-down and knock-in techniques. Single protein manipulations, while very important, cannot be expected to reliably predict phenotypic changes in light of growing awareness of complex feedback regulation of gene-protein networks. We plan to explore these manipulations in subsequent investigation, until now having focused on phenotypic behaviors of seven different cancer lines in contrast to controls.

Cancers are notorious for their ability to evade metabolic regulation [20] and therapeutic use of metabolic inhibitors has been avoided until recently. Specifically targeted pharmacologic inhibitors such as dichloroacetate [21] and 2-deoxyglucose [22,23], the latter originally proposed as a metabolic inhibitor in 1960 [24], have shown promise in the inhibition of cancer growth, although toxicity to normal cells has been a limitation. It is worthwhile to ask whether less toxic metabolic regulation of cancer cells is possible in light of current thinking about cancer evolution, development and progression [25,20]. We have previously outlined a general hypothesis bearing on this [6] and proposed, along with others [26] that human evolutionary adaptations to starvation render normal cells tolerant of flexible nutrient sources, including ketone bodies and fatty acids.

On the other hand the initiation of a primary event transforming a normal cell into a pre-malignant phenotype is followed by a long period of subsequent adaptive cellular response to a local hypoxic, nutrient-deprived microenvironment. During this interval of abnormal cellular growth multiple adaptations eventually result in the cancer phenotype[27]. A typical response to the hypoxic environment, for example, is expression of insulin-independent GLUT1's [25,28] triggered by HIF 1α [29] insuring maximum glucose uptake for glycolytic ATP generation. Solid cancers require at least 30 to 35 generational doublings before they attain a clinically detectable size of 1–2 cm diameter (assuming average mammalian cell diameter of 10 microns) [30] over durations of 3 to 15 years. The abundance and low cost of grain characteristic of efficient modern agriculture has made sustained ketosis an unfamiliar internal metabolic environment for many people living in developed countries [6]. It follows that in large cohorts of individuals, sustained ketosis is not a familiar microenvironment for newly evolving abnormal cells, on the path to malignancy. Nevertheless, our normal tissues remain adapted to these microenvironments as shown by the absence of toxicity in experimental fasting of morbidly obese individuals in the 1960's[31]. In human cancers in the developed world, and in the absence of sustained ketosis as a selective pressure over durations of 30 or more doubling times, a diversity of evolutionary responses should then be expected including accidental vulnerabilities and survival adaptations. One response that would correspond to an accidental vulnerability to ketosis would be over-expression of UCP2 with intact TCA enzymes.

## Conclusion

We have shown that acetoacetate added to glucose medium causes variable but parallel reductions of ATP concentration and cell growth in seven human cancer cell lines. The effect lies along a continuum with control fibroblasts at one extreme in which neither cell growth is inhibited nor is ATP concentration reduced. The effect is consistent with an "inefficient" Randle cycle due to over-expression of uncoupling protein 2, seen only in the cancer lines, and is plausible in light of current models of a transformed cell's adaptive microscopic development and progression toward the cancer phenotype. More work must be done to prove metabolic inhibition by this mechanism. If proven, the results speak to the hypothesized potential for ketogenic diets as non-toxic therapeutic strategies, as adjuvants to standard therapies, and to multimodality therapies to improve the control of malignant disease.

## Competing interests

The authors have no conflicting or competing interests to declare in the conception, evolution, or execution of this research.

## Authors' contributions

EJF conceived the study, directed its primary design and coordination, performed much of the data analysis, and drafted and revised the manuscript; AM grew and counted the cell cultures, performed the ATP assays, performed substantial data analysis and participated in writing the Methods section of the manuscript; EVQ coordinated execution of the study, contributed to important aspects of the research direction, and made important revisions to the manuscript; JMS performed the UCP2 assay and wrote the methods for this aspect of the work; RDF coordinated execution of the study, contributed to important aspects of the research direction, and made important revisions to the manuscript. All authors read and approved the final manuscript.
